# Case Report: Exposure to Respirable Crystalline Silica and Respiratory Health Among Australian Mine Workers

**DOI:** 10.3389/fpubh.2022.798472

**Published:** 2022-06-14

**Authors:** Krassi Rumchev, Dong Van Hoang, Andy Lee

**Affiliations:** ^1^School of Population Health, Curtin University, Perth, WA, Australia; ^2^Department of Epidemiology and Prevention, Center for Clinical Sciences, National Center for Global Health and Medicine, Tokyo, Japan

**Keywords:** miners, silica, occupational exposure, respiratory symptoms, Australia

## Abstract

Occupational exposure to respirable crystalline silica (RCS) is common in a range of industries, including mining, and has been associated with adverse health effects such as silicosis, lung cancer, and non-malignant respiratory diseases. This study used a large population database of 6,563 mine workers from Western Australia who were examined for personal exposure to RCS between 2001 and 2012. A standardized respiratory questionnaire was also administered to collect information related to their respiratory health. Logistic regression analyses were performed to ascertain the association between RCS concentrations and the prevalence of respiratory symptoms among mine workers. The estimated exposure levels of RCS (geometric mean 0.008mg/m^3^, GSD 4.151) declined over the study period (*p* < 0.001) and were below the exposure standard of 0.05 mg/m^3^. Miners exposed to RCS had a significantly higher prevalence of phlegm (*p* = 0.017) and any respiratory symptom (*p* = 0.013), even at concentrations within the exposure limit. Miners are susceptible to adverse respiratory health effects at low levels of RCS exposure. More stringent prevention strategies are therefore recommended to protect mine workers from RCS exposures.

## Introduction

Crystalline silica is one of the most abundant minerals found in the earth's crust and it is used in many products across a variety of industries and occupations including underground mining. During mechanical processes such as crushing, cutting, drilling, or grinding of natural stone, or man-made products that contain silica, workers generate silica dust commonly referred to as respirable crystalline silica (RCS) (1). Silica dust particles with size from 0.01 to 100 μm in diameter are a significant health concern (2). When inhaled, RCS can penetrate deep into the lungs and cause irreversible lung damage including chronic bronchitis, chronic obstructive pulmonary disease (COPD), emphysema, silicosis, and silico-tuberculosis (3, 4). According to a study by Steenland (3), RCS can remain in the lungs even if the exposure is stopped and may continue to adversely affect its functions.

The Australian standard for workplace exposure to silica is 0.05mg/m^3^ (50 μg/m^3^) over an 8-h working day, for a 5-day working week (5). It is consistent with the international recommended exposure limit of 0.05 mg/m^3^ as a time-weighted average for up to 10 h per day during a 40-h week (1). However, epidemiological studies have indicated that such occupational standard is not sufficient to protect against chronic silicosis (3).

Around 3.2 million workers are exposed to RCS in the European Union and 1.7 million in the United States (6). In 2011, approximately 587,000 Australian workers were exposed to silica dust in the workplace, of whom 5,758 will consequently develop lung cancer over the course of their life (7, 8).

In recent years, occupational exposure levels of RSC have decreased worldwide. A recent review found overall decreases in exposure levels of respirable dust and respirable quarts for the European minerals industry during a 15-year period (2002–2016) (9). Downward trends in quartz exposure for total dust and RCS were reported in China from 1950 to 1987 (10). Similarly, an Australian study demonstrated an overall downward trend in silica exposure of about 8% per year for the period 1986–2014 (11). The reduction of RCS exposure levels in most developed countries during the last century resulted in dramatic decreases in morbidity and mortality from silica dust associated health effects (12). Despite this, chronic obstructive pulmonary disease (COPD) remains a health issue in workers exposed to RCS. The exposure can lead to airflow obstruction in the absence of radiological signs of silicosis, while the association between cumulative silica dust exposure and airflow obstruction can be independent of silicosis (12). Therefore, it is likely that certain properties of silica dust can cause COPD that may precede or be independent of silicosis development.

The objective of the present case study was to ascertain the association between exposures to RCS and the prevalence of respiratory symptoms among Australian miners. The novelty of the present study was the exposure assessment of low levels of RCS in relation to respiratory symptoms among a large population of miners over a 12-year period. The research findings may have important implications for the development of further prevention strategies to protect mine workers from exposure to RCS.

## Materials and Methods

### Study Protocol and Study Population

This study used an industry-wide occupational exposure database for respirable mineral dust from the Department of Mines and Petroleum (DMP). In Western Australia, mining companies were required to conduct exposure assessments of certain occupational hazards, including RCS, and report the findings to the DMP. The occupational hygiene data were directly entered into the CONTAM database by the companies. The available dataset consisted of 6,563 mine workers for the period 2001–2012 who were involved in 192 mining related activities. For the purpose of this study, these activities were classified into three occupation groups namely “manager,” “surface production and services,” and “underground mining.” The first group represented mine management occupations including managers, operation supervisors, superintendents, and engineers. The second group consisted of occupations in which workers were not exposed or had limited exposure to dust-generating activities including geologists, mobile plant operators, electricians, and mechanics. The third group comprised mine workers who were involved in underground mining production activities such as drilling, blasting, and loading. The type of mines included gold, nickel, iron, and other minerals. The dataset provided by DMP was summarized by calendar year, occupation, age, sex, shift length, protective mask-wearing, and current smoking status (in the last 3 months).

### Exposure Estimation and Outcome Assessment

Measurements of personal exposure to respirable particles were conducted by the mining companies. During each year of survey and data collection, for practical purposes, miners with similar exposure profiles were grouped together by the companies using their list of employees, before samples were randomly selected from each similar exposure group. The method used to sample respirable dust (RES) was conducted in accordance with the Australian Standard for sampling and gravimetric determination of respirable dust in workplace atmospheres (AS2985-2009), which follows the International Standard ISO 7708:1995, Air quality—Particle size fraction definitions for health-related sampling. The air samples were collected in the breathing zone of mine workers for 8 h using cyclones for gravimetric dust sampling. The content of RCS was performed by infrared spectroscopy following the NHMRC standard method (13).

In addition to hygiene data, regular respiratory health surveillance was also undertaken by the mining companies using a respiratory questionnaire, lung function test, an audiometric (hearing) test and in some cases, a chest x-ray. However, only data from the respiratory questionnaire were provided to the present study. The survey comprised questions about current respiratory symptoms occurring in the last 3 months, including, “cough” (usually cough first in the morning, during the day or at night), “phlegm” (usually bring up phlegm first in the morning, during the day or at night), “wheeze” (ever experience of the chest sounding wheezy or whistling), “breathlessness” (either short of breath at rest or on activity), and “any respiratory symptom” (having at least one of the above symptoms. Further details on the health data collection have been described previously (14). The CONTAM and Health database were linked, using each worker's unique identifier number (14). Our analysis was based on estimated current exposures to RCS and RES and current respiratory symptoms.

### Statistical Analysis

RCS and RES concentrations were presented as geometric mean (GM) with geometric standard deviation (GSD). Arithmetic means (AM) and standard deviations (SD) were used to describe other continuous variables and percentages were used for categorical variables. The total geometric means of RCS and RES concentrations were tabulated and compared with respect to the status of each respiratory symptom (cough, phlegm, breathlessness, wheeze, or any respiratory symptom). The prevalence of each and any respiratory symptoms was calculated annually as described before (14). *T*-test and Wilcoxon sign test were used for comparison of arithmetic and geometric means, respectively, whereas Chi-squared test was applied to compare the prevalence of respiratory symptoms.

To ascertain the association between RCS concentration levels and the prevalence of respiratory symptoms, logistic regression models were fitted, with adjusted odds ratios (OR) and their corresponding 95% confidence intervals (CI) for quantifying the observed association, accounting for the effects of plausible confounding factors including age, sex, occupation, mask wearing, work shift length, smoking status, and RES. A logarithmic transform was applied to RCS prior to logistic regression analysis due to its skewed distribution. All statistical analyses were performed in RStudio for Windows (version 3.2.4).

## Results

This study used a large population data set of 6,951 observations from Western Australian mine workers for the period 2001–2012. Of the total cumulative number of mine workers (6,951), 6,563 were monitored once, 178 were monitored twice, eight were monitored three times, and two were monitored four times. The mean age of the study population was 35.8 years (SD 10.9). Overall, most workers were men (90.4%) and worked a long shift of 12 h (79.7%). Most of the study subjects were engaged in underground mining activities (52.4%), followed by service production (29.3%) and 18.2% were managers. Approximately 84% of mine workers did not wear a protective mask during dusty activities and the majority (65.5%) were non-smokers (Table 1).

**Table 1:** Study population characteristics.

**Characteristics**	**2001**	**2002**	**2003**	**2004**	**2005**	**2006**	**2007**	**2008**	**2009**	**2010**	**2011**	**2012**	**Total**
*N*	410	558	512	417	494	444	561	778	485	689	707	896	6,951
Age, mean (SD)	35.9 (9.9)	36.7 (10.3)	36.8 (10.4)	34.4 (10.0)	34.2 (10.8)	36.8 (11.2)	36.7 (10.9)	35.5 (10.8)	36.1 (11.2)	35.9 (11.1)	35.2 (11.6)	35.7 (11.3)	35.8 (10.9)
Sex, *n* (%)													
Female	27 (6.6)	35 (6.3)	38 (7.4)	37 (8.9)	36 (7.3)	44 (9.9)	56 (10.0)	90 (11.6)	47 (9.7)	72 (10.4)	80 (11.3)	102 (11.4)	664 (9.6)
Male	383 (93.4)	523 (93.7)	474 (92.6)	380 (91.1)	458 (92.7)	400 (90.1)	505 (90.0)	688 (88.4)	438 (90.3)	617 (89.6)	627 (88.7)	794 (88.6)	6,287 (90.4)
Occupation, *n* (%)													
Manager	80 (19.5)	95 (17.0)	106 (20.7)	66 (15.8)	92 (18.6)	76 (17.1)	110 (19.6)	154 (19.8)	79 (16.3)	117 (17.0)	127 (18.0)	165 (18.4)	1,267 (18.2)
Surface production	106 (25.9)	133 (23.8)	120 (23.4)	101 (24.2)	146 (29.6)	148 (33.3)	160 (28.5)	225 (28.9)	155 (32.0)	235 (34.1)	231 (32.7)	279 (31.1)	2,039 (29.3)
Underground mining	224 (54.6)	330 (59.1)	286 (55.9)	250 (60.0)	256 (51.8)	220 (49.5)	291 (51.9)	399 (51.3)	251 (51.8)	337 (48.9)	349 (49.4)	452 (50.4)	3,645 (52.4)
Mask wearing, *n* (%)													
No	365 (89.0)	496 (88.9)	416 (81.2)	348 (83.5)	386 (78.1)	394 (88.7)	494 (88.1)	632 (81.2)	394 (81.2)	566 (82.1)	576 (81.5)	750 (83.7)	5,817 (83.7)
Yes	45 (11.0)	62 (11.1)	96 (18.8)	69 (16.5)	108 (21.9)	50 (11.3)	67 (11.9)	146 (18.8)	91 (18.8)	123 (17.9)	131 (18.5)	146 (16.3)	1,134 (16.3)
Shift length, *n* (%)													
10 h	103 (25.1)	170 (30.5)	121 (23.6)	114 (27.3)	82 (16.6)	88 (19.8)	147 (26.2)	185 (23.8)	100 (20.6)	106 (15.4)	75 (10.6)	121 (13.5)	1,412 (20.3)
12 h	307 (74.9)	388 (69.5)	391 (76.4)	303 (72.7)	412 (83.4)	356 (80.2)	414 (73.8)	593 (76.2)	385 (79.4)	583 (84.6)	632 (89.4)	775 (86.5)	5,539 (79.7)
Smoking status, *n* (%)													
Non-smoker	249 (60.7)	345 (61.8)	320 (62.5)	262 (62.8)	323 (65.4)	285 (64.2)	362 (64.5)	494 (63.5)	329 (67.8)	472 (68.5)	500 (70.7)	613 (68.4)	4,554 (65.5)
Smoker	161 (39.3)	213 (38.2)	192 (37.5)	155 (37.2)	171 (34.6)	159 (35.8)	199 (35.5)	284 (36.5)	156 (32.2)	217 (31.5)	207 (29.3)	283 (31.6)	2,397 (34.5)

In total, 29.8% of mine workers reported at least one respiratory symptom, with cough (14.7%) being the most common symptom, followed by wheeze (12.8%), phlegm (10.6%), and breathlessness (9.2%). Table 2 shows that the prevalence of respiratory symptoms was significantly higher among older mine workers than their younger colleagues. Elevated exposure levels to RCS were recorded for miners who reported respiratory symptoms when compared to those with no such symptoms, and the differences were significant for all symptoms except for breathlessness.

**Table 2:** Distribution of RCS and RES concentrations (mg/m^3^) and age (years) of miners by the status of respiratory symptom.

**Respiratory symptom**	**No**	**Yes**	***p***
Cough			
*n*	5,931	1,020	
Age^1^	35.5 (10.9)	37.6 (10.8)	<0.001
RCS^2^	0.008 (4.234)	0.009 (4.019)	0.003
RES^2^	0.256 (2.672)	0.274 (2.614)	0.007
Phlegm			
*N*	6,213	738	
Age^1^	35.6 (10.9)	37.2 (11.1)	<0.001
RCS^2^	0.008 (4.225)	0.009 (4.007)	0.002
RES^2^	0.257 (2.669)	0.271 (2.623)	0.079
Breathlessness			
*N*	6,309	642	
Age^1^	35.4 (10.7)	40.3 (11.3)	<00.001
RCS^2^	0.008 (4.270)	0.008 (3.588)	0.48
RES^2^	0.262 (2.683)	0.231 (2.460)	0.004
Wheeze			
*N*	6,062	889	
Age^1^	35.7 (10.9)	36.7 (11.1)	0.014
RCS^2^	0.008 (4.223)	0.009 (4.074)	0.026
RES^2^	0.259 (2.669)	0.260 (2.630)	0.69
Any symptom			
*N*	4,908	2,043	
Age^1^	35.1 (10.7)	37.5 (11.1)	<00.001
RCS^2^	0.007 (4.280)	0.008 (4.015)	0.001
RES^2^	0.258 (2.692)	0.260 (2.597)	0.38

*RCS, respirable crystalline silica; RES, respirable dust.*

1 *arithmetic mean (SD), P-values were obtained from two-sample t-test;*

2 *geometric mean (GSD), P-values were obtained from Kruskal Wallis test*.

**Figure 1:**
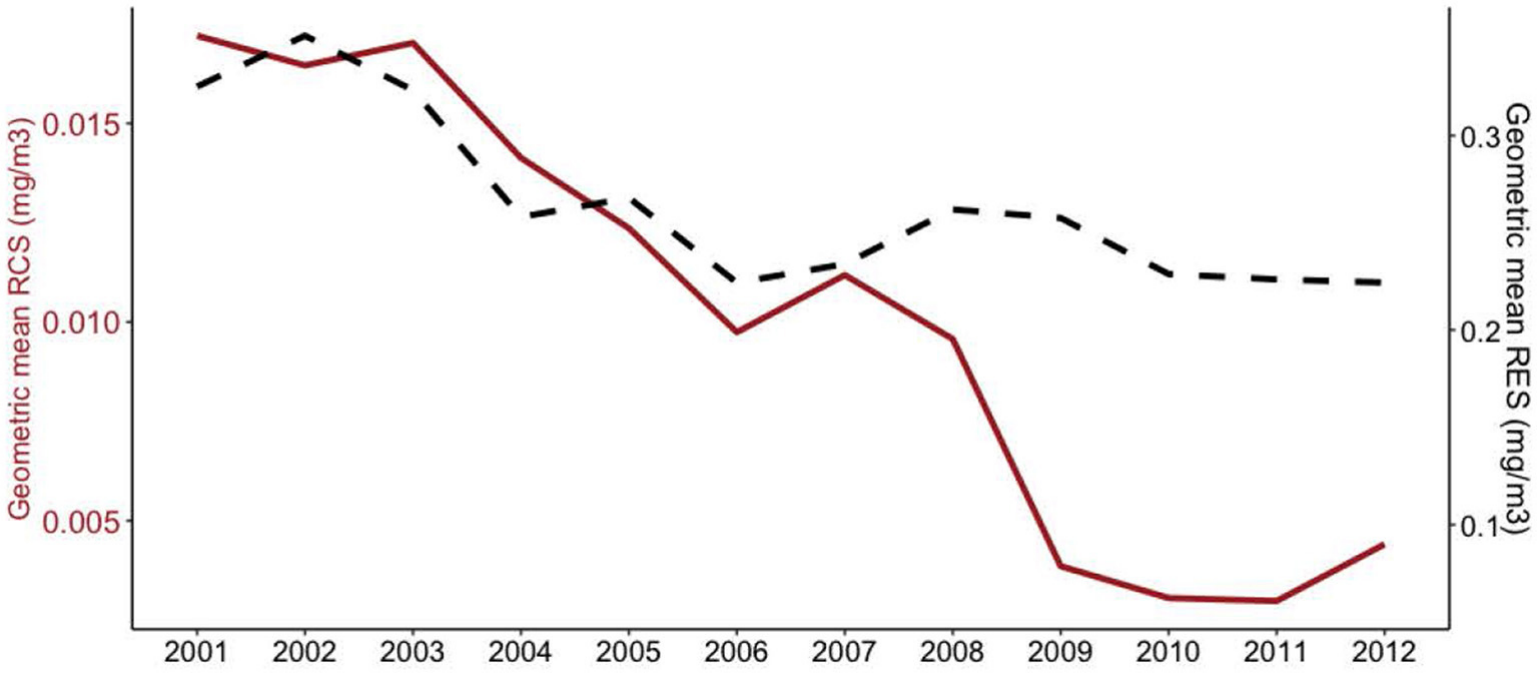
Time trend of GM of RCS and RES concentrations.

The GM of RCS concentration recorded over the study period was 0.008 (GSD 4.151) mg/m^3^ with the range 0.001–11.0 mg/m^3^ and the GM of RES concentration recorded over the study period was 0.258 (GSD 2.640) mg/m^3^ with the range 0.033–35.000 mg/m^3^. As shown in Figure 1, the study observed a steady decline (*p* < 0.001) in RCS and RES exposure levels between 2001 and 2012. Substantial variability in RCS exposure was evident between the three occupation categories, with underground miners having a significantly higher exposure level (0.009 mg/m^3^, GSD 4.087) than managers (0.008 mg/m^3^, GSD 3.878) and surface production workers (0.006 mg/m^3^, GSD 4.326).

Significantly higher (*p* < 0.01) prevalence of phlegm, cough, and any respiratory symptom were found for male than female mine workers (Supplementary Table). As expected, higher frequency of cough, phlegm, wheeze, breathlessness, and any respiratory symptom were reported by smokers than their non-smoking counterparts and the differences were significant (Supplementary Table). The study found that miners who wore a protective mask during work had significantly (*p* = 0.016) less phlegm (8.6%) when compared to others without any respiratory protection (11.0%). No significant difference was generally found in the prevalence of respiratory symptoms between the three occupation groups, apart from phlegm which underground miners reported a higher rate (12.1%) than managers (9.4%) and surface production workers (8.7%).

Results from multivariate logistic regression analyses, summarized in Table 3, show significant associations between the prevalence of phlegm, wheeze and any respiratory symptom and low exposure to RCS. Specifically, miners exposed to RCS were at risk of increased prevalence of phlegm with adjusted OR 1.06 (95% CI 1.00–1.130), wheeze with adjusted OR 1.05 (1.00–1.11), and any respiratory symptom with adjusted OR 1.06 (95% CI 1.020–1.10). The differences between males and females were significant only for phlegm and any respiratory symptom after controlling for other confounding factors. The logistic regression models further confirmed the significant adverse effects of age and smoking, whereas occupation, shift length, and mask-wearing had little association with the prevalence of respiratory symptoms.

**Table 3:** Association of log RCS with respiratory symptoms.

**Respiratory symptoms**	***N* (%)**	**Odds Ratio (95% CI)**		
		**Model 1**	**Model 2**	**Model 3**
Cough	1,020 (14.7)	1.06 (1.02, 1.12)	1.04 (0.99, 1.09)	1.05 (0.99, 1.11)
Phlegm	738 (10.6)	1.08 (1.03, 1.14)	1.06 (1.00, 1.12)	1.06 (1.00, 1.13)
Breathlessness	642 (9.2)	1.01 (0.95, 1.07)	1.02 (0.96, 1.08)	1.05 (0.98, 1.12)
Wheeze	889 (12.8)	1.05 (1.00, 1.10)	1.03 (0.98, 1.09)	1.05 (1.00, 1.11)
Any symptom	2,043 (29.4)	1.06 (1.02, 1.09)	1.04 (1.00, 1.08)	1.06 (1.02, 1.10)

*Log (RCS) was used instead of RCS due to its skew distribution.*

*Model 1, unadjusted; Model 2, adjusted for age (years), sex, occupation, mask wearing, smoking status, and shift length; Model 3, adjusted for RES plus covariates included in Model 2*.

## Discussion

The frequent exposure to RCS in workplaces has placed a considerable number of occupational populations at risk of work-related diseases. It has been acknowledged that diseases arising from exposure to RCS share some common features such as respiratory symptoms, disability, and silicosis. In the early stage of silicosis, symptoms are usually mild and include cough, phlegm, and progressive shortness of breath (15).

The present study assessed exposure to airborne RCS and respiratory health among a large population of mine workers in Western Australia between 2001 and 2012. The outcomes showed that exposure to RCS has declined steadily over the study period, which is consistent with previous studies conducted in the USA, Europe, China, and Canada (9, 10, 16, 17). This could have resulted from the enforcement of improved control measures by governments including in Australia (11). Our observed annual RCS concentrations were below the exposure limit of 0.05mg/m^3^, and the downward trend in exposure levels was in line with the decrease in the prevalence of respiratory symptoms during the study period.

Despite that mine workers were exposed to RCS at levels well below the accepted limit of 0.05 mg/m^3^, they experienced a significantly higher prevalence of phlegm and any respiratory symptom. This finding raises the concern that mine workers involved in silica-associated jobs are susceptible to adverse respiratory health effects even at low levels of RCS exposure. Indeed, our finding agrees with several epidemiological studies that suggested the current occupational standards are insufficient to protect against silica-related respiratory illnesses (18–29). In a study conducted in Iran, 37% of exposed workers to silica reported phlegm, 18.5% had a cough, 14.8% had shortness of breath, and 7.4% had wheeze (30). Similar findings were reported in other studies (3, 15, 18, 31). Hnidzo and colleagues (12) found that chronic lower levels of silica exposure may lead to the development of emphysema and chronic bronchitis that in turn can lead to airflow obstruction, even in the absence of radiological signs of silicosis. A significant increase in mortality from non-malignant respiratory diseases such as chronic bronchitis, emphysema, and asthma for silica-exposed workers has also been previously reported (19–21). Moreover, previous research demonstrated that the excess lifetime risk of lung cancer at the current silica standard (about 0.05 mg/m^3^ for the cristobalite form of silica) is estimated to be over 5% (22). It is considered that even exposures at 0.01 mg/m^3^ may cause an unacceptable risk for silicosis (22–29).

Smoking played a significant role in the prevalence of respiratory symptoms for mine workers in this study, consistent with previous findings (15, 32). A recent study (33) also confirmed the interaction effects between smoking and occupational dust exposure on respiratory health. Smoking can subsequently increase the susceptibility of mine workers to dust particles, indicating the need for an increased focus on the treatment of tobacco dependence in the work environment.

Despite previous reports that no gender differences existed in the prevalence of respiratory diseases regarding silica exposure (34, 35) this study found a significantly higher frequency of some respiratory symptoms among male mine workers than their female colleagues. Although there is inconsistency in the reported study findings concerning sex differences and disease outcomes, it is apparent that there is a risk for both sexes.

The use of administrative databases in the current study had some strengths but was also associated with limitations. The lack of involvement by the investigators in the data collection process is acknowledged as a study limitation. The use of self-reported respiratory symptoms without medical diagnoses posed another limitation. Nevertheless, self-report data from the respiratory health survey were used in conjunction with the quantitative RCS measurements and other information through record matching and data linkage to reduce bias and inaccuracy (36). The study also acknowledges that length and level of RCS exposure, and other confounding factors than those considered in the logistic regression model, such as diesel particulate matter, coal dust, and metalliferous mine dust not available for the current study, could affect the association between respiratory symptoms and RCS. Indeed, the weakness of the cross-sectional study design and single-point exposure estimation made any conclusion about the apparent association difficult to establish.

This study demonstrated that although the airborne levels of RCS are brought into compliance with the exposure limit, it is recommended that mine workers should be further protected from inhaling silica and occupational interventions to reduce airborne silica dust exposure should be prioritized on high exposure tasks.

## Conclusion

This study is consistent with existing evidence (12, 15) that suggested adverse respiratory health effects for mine workers even at levels of RCS exposure within the limit. It is vital for developing further prevention strategies to protect mine workers from exposure to RCS in Australia and elsewhere.

## Data Availability Statement

The raw data supporting the conclusions of this article will be made available by the authors, without undue reservation.

## Ethics Statement

The studies involving human participants were reviewed and approved by the Department of Mines and Petroleum, Perth, Western Australia. The patients/participants provided their written informed consent to participate in this study.

## Author Contributions

KR and AL contributed to the conception and presentation of this report. DH conducted the data analysis. AL provided expertise and interpretation of the statistical results. All authors contributed to the manuscript writing, and have read and agreed to the published version of the manuscript.

## Conflict of Interest

The authors declare that the research was conducted in the absence of any commercial or financial relationships that could be construed as a potential conflict of interest.

## Publisher's Note

All claims expressed in this article are solely those of the authors and do not necessarily represent those of their affiliated organizations, or those of the publisher, the editors and the reviewers. Any product that may be evaluated in this article, or claim that may be made by its manufacturer, is not guaranteed or endorsed by the publisher.
