# Supplementary material for: Case Report: Exposure to Respirable Crystalline Silica and Respiratory Health Among Australian Mine Workers
Source: Front Public Health. 2022 Jun 14;10:798472. doi: 10.3389/fpubh.2022.798472 (PMC9234445; doi:10.3389/fpubh.2022.798472)
Supplement: Supplementary file 1 [file Table_1.pdf]

Supplementary Table Prevalence of respiratory symptoms according to potential confounders

	Respiratory symptoms, n (%)				
Potential confounders	Cough	Phlegm	Breathlessness	Wheeze	Any symptom
Sex					
Female	74 (11.2)	41 (6.2)	60 (9.1)	71 (10.7)	160 (24.2)
Male	945 (15)	696 (11.1)	582 (9.3)	818 (13)	1882 (29.9)
p-value	0.01	0	0.937	0.11	0.002
Occupation					
Manager	211 (16.7)	118 (9.4)	130 (10.3)	178 (14.1)	390 (30.9)
Surface mining	262 (12.6)	181 (8.7)	165 (8)	267 (12.9)	563 (27.2)
Underground mining	546 (15.1)	438 (12.1)	347 (9.6)	444 (12.3)	1089 (30.1)
p-value	0.003	0	0.043	0.239	0.027
Shift length					
10 hours	207 (14.3)	163 (11.2)	142 (9.8)	177 (12.2)	444 (30.6)
12 hours	812 (14.8)	574 (10.4)	500 (9.1)	712 (13)	1598 (29.1)
p-value	0.653	0.414	0.452	0.467	0.274
Mask wearing					
Not worn	856 (14.9)	633 (11)	548 (9.5)	733 (12.7)	1710 (29.7)
Worn	163 (13.6)	104 (8.7)	94 (7.9)	156 (13.1)	332 (27.8)
p-value	0.293	0.022	0.081	0.801	0.194
Smoking					
Current smoker	640 (26.7)	427 (17.8)	285 (11.9)	472 (19.7)	1040 (43.4)
Non-smoker	379 (8.3)	310 (6.8)	357 (7.8)	417 (9.2)	1002 (22)
p-value	0	0	0	0	0

p-value obtained from Chi-squared test
